# Computer simulation study of nutrient-driven bacterial biofilm stratification

**DOI:** 10.1098/rsif.2023.0618

**Published:** 2024-06-26

**Authors:** Francisco Javier Lobo-Cabrera, María del Río Herrero, Fernando Govantes, Alejandro Cuetos

**Affiliations:** ^1^ Center for Nanoscience and Sustainable Technologies (CNATS) and Department of Physical, Chemical and Natural Systems, Pablo de Olavide University, Sevilla, Spain; ^2^ Departamento de Biología Molecular e Ingeniería Bioquímica, Centro Andaluz de Biología del Desarrollo (Universidad Pablo de Olavide, Consejo Superior de Investigaciones Científicas y Junta de Andalucía) and Universidad Pablo de Olavide, Sevilla, Spain

**Keywords:** computer simulation, biofilm development, individual-based model, biofilm stratification

## Abstract

Here, employing computer simulation tools, we present a study on the development of a bacterial biofilm from a single starter cell on a flat inert surface overlaid by an aqueous solution containing nutrients. In our simulations, surface colonization involves an initial stage of two-dimensional cell proliferation to eventually transition to three-dimensional growth leading to the formation of biofilm colonies with characteristic three-dimensional semi-ellipsoids shapes. Thus, we have introduced the influence of the nutrient concentration on bacterial growth, and calculated the cell growth rate as a function of nutrient uptake, which in turn depends on local nutrient concentration in the vicinity of each bacterial cell. Our results show that the combination of cell growth and nutrient uptake and diffusion leads to the formation of stratified colonies containing an inner core in which nutrients are depleted and cells cannot grow or divide, surrounded by an outer, shallow crust in which cells have access to nutrients from the bulk medium and continue growing. This phenomenon is more apparent at high uptake rates that enable fast nutrient depletion. Our simulations also predict that the shape and internal structure of the biofilm are largely conditioned by the balance between nutrient diffusion and uptake.

## 1. Introduction

Biofilm formation is an ordered regulated process, akin to multi-cellular development in complex organisms. The typical biofilm formation sequence involves initial interaction with the surface that leads to weak, reversible binding. Commitment to surface colonization is hallmarked by the transition to irreversible binding, a stronger form of interaction mediated by adhesion factors recruited on the cell surface. The biochemical nature of such adhesion factors varies from species to species, ranging from extracellular polysaccharides to a variety of adhesion proteins or even complex proteinaceous appendages [1,2]. Transition to irreversible attachment is concomitant to the loss of flagella when present and the onset of growth and cell division, usually by binary fission [3]. At this stage, cell proliferation as a surface-associated flat monolayer concomitant to the production of the components of the biofilm matrix leads to the formation of microcolonies [4]. In a submerged biofilm, in which nutrients are carried in the overlying liquid phase, this bidimensional structure grants all the cells access to the external boundary of the biofilm and nutrients. Biofilm microcolonies evolve further by piling up additional cell layers to become three-dimensional mature biofilm colonies and eventually acquire complex shapes and internal structures [5–7].

Three-dimensional biofilm development is conditioned by multiple factors. Specifically, environmental factors, such as nutrient availability, are critical determinants of the structure and properties of the biofilm throughout its developmental process. Accordingly, high nutrient concentration and better access of the entire biofilm to nutrients promotes greater robustness, thickness and activity [8]. In contrast, restricted access to nutrients often favours cell detachment, leading to centrally located voids in sufficiently large clusters of cells. In this situation, growth-rate gradients along the biofilm were observed [9], suggesting non-homogeneous distribution of nutrients in the biofilm. Other authors have pointed out that the establishment of stable gradients of nutrients generate different local micro-habitats, provoking physiological stratification and heterogeneity in biofilms to determine the internal spatial organization, both in monospecies and mixed-species biofilms [10]. Interestingly, a similar situation occurs in tumours, where nutrient depletion in the inner regions leads to the restricted location of dividing cells near the outer surface, resulting in reduced overall growth rate [11].

Given the economic relevance of biofilms, the breadth of views and tools in biofilm studies is not surprising. Specifically, over the last two decades, computer modelling and computer simulation have gained importance in the study of biofilm development [12–14]. Among the models used, one of the most promising families are those known as the individual-based models (IbM). In this approach, the features of the cell community (from microcolonies to mature biofilm colonies) are described as emergent properties derived from the interactions of the individual bacterial cells [14–16]. This point of view is very similar to that used in the field of molecular simulation, especially in the molecular dynamics field. Thus, this set of techniques could be classified under the common umbrella of cell simulation.

Among the IbM models, those dedicated to exploring the early development of two-dimensional biofilms [17–19], or the transition from two-dimensional to three-dimensional colonies [7,20,21], stand out. In these conditions, all cells have equal access to nutrients and the key factors controlling microcolony development are competition between cell growth and diffusion [17,19], and mechanical stress within the biofilm [18,22]. In IbM models focused on the description of three-dimensional biofilms, the distribution of nutrients is a key factor modulating bacterial cell elongation and division, to eventually condition the internal structure of the colony. This was pointed out early on by Picioreanu *et al.* [23], who introduced nutrient diffusion and uptake coupled to cell division, as well as the interaction and mechanical relaxation of the cells within the colony in their model. This and subsequent works [24–29] show how competition between nutrient diffusion and uptake generates concentration gradients that influence the internal structure and shape of the biofilm colonies. One of the limitations of these models is that they do not usually take into account the non-spherical shape often found in bacterial cells, which may be relevant to explain some structural features of biofilms.

Recently, we have developed a computational model of bacterial biofilm growth. Our model, with the characteristics of an IbM, includes rod-shaped bacterial cells, binary fission as the mechanism of cell division, the steric interaction between the bacterial cells and the consequent Brownian diffusion. We have used this model to investigate early biofilm development, restricted to the two-dimensional stage. Our work highlights the importance of competition between diffusion and cell growth to determine colony shape and internal architecture. Thus, in situations where growth is faster than typical cell diffusion, compact colonies are obtained; in the opposite situation, fast cell diffusion on the substrate leads to the formation of open swarms in which most cells are not in contact with each other [17,19]. We have also studied the relevance of the presence of polymers in the medium, which can favour colony aggregation and compaction by different mechanisms, depending on the size and concentration of the polymer [30].

Since the above studies focused on two-dimensional colony growth, in which all bacteria have equal access to nutrients, the dependence of the growth rate on nutrient concentration and uptake rate was not considered. The processes by which the microcolony evolves from two-dimensional to three-dimensional were also not considered. In this paper, we present an extension of our previous work. In keeping with the general philosophy of our preceding studies, we introduce the transition from two-dimensional colonies and the subsequent growth of three-dimensional biofilms. Accordingly, the extension of our model discussed here includes the dependence of the growth rate on the local nutrient concentration for each individual cell. This concentration is modulated by nutrient diffusion from the bulk medium. We aim to study the relationship between biofilm structure and shape with nutrient uptake and diffusion while considering aspects observed in our previous studies such as competition between cell growth and diffusion. The results of this study can be relevant to understanding the aspects of the internal structure of biofilms that are not amenable to experimental analysis.

## 2. Methods

**Figure 1.**
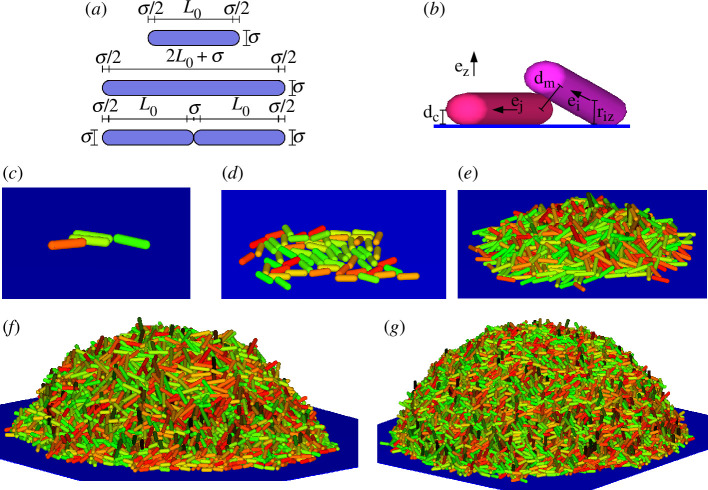
Details of the model. (*a*) Representation of the geometrical parameters of the bacterial model. The cartoon shows a particle in the the initial state with length $L_{0}$, a predivisional particle with the maximum length and the two products of the division, which correspond to the initial state for the following cycle. (*b*) Distances and vectors are used in the calculation of the interaction between two particles, and between particles and substrate (see text for details). (*c*–*g*) Sequence in the growth of a simulated biofilm in the case of $D_{N}^{*}=1000$ and $K_{s}^{*}=10$. The number of particles in each snapshot is from (*c*) to (*g*) 4, 64, $10^{3}$, $10^{4}$ and $3\times 10^{4}$.

The model used here is an extension of that previously developed and presented by Acemel *et al.* [17], introducing a third dimension and coupling growth rate with nutrient uptake. A detailed explanation of the algorithm used is given below. An outline in pseudocode format summarizing the algorithm used and a table listing the quantities and parameters defined in this section are provided in the electronic supplementary material. Here, a biofilm colony is assumed to evolve on an infinite inert flat substrate at z=0 overlaid by an infinite nutrient-containing liquid phase. The bacterial cells forming the biofilm are modelled as spherocylinders. This is a three-dimensional body made up of a cylinder of initial length $L_{0}$ with hemispheres of diameter σ located at both ends (figure 1*a*). Therefore, the initial aspect ratio is $L_{0}^{*}=L_{0}/\sigma +1$ will be considered as the unit of length throughout this work. In order to mimic the characteristics of the bacterial species *Pseudomonas putida*, $L_{0}^{*}$ was set as $L_{0}^{*}=2.6$ [31,32]. These spherocylindrical particles grow by extending the length of the cylinder over time. When the particles double their initial length ($L^{*}=2L_{0}^{*}$) they divide into two new particles of length $L_{0}^{*}$, displaying the same initial orientation as the initial particle (figure 1*a*). In contrast with the scenario proposed by Acemel *et al.* [17], here such growth is not constant, but dependent on the local nutrient concentration according to the equation


(2.1) $$v_{i}=\frac{v_{eff}\cdot \mu_{i}(x,y,z,t)}{L_{i}^{*}},$$

where $v_{i}$ represents the growth rate of each bacterial cell i at time *t*. $L_{i}^{*}=L_{i}/\sigma +1$ is the instantaneous aspect ratio of bacterium i. $v_{eff}$ accounts for the fraction of nutrients devoted to biomass growth. This factor has been introduced to take into account that cells may need substrate to maintain other functions in addition to the growth and division process. Other authors have proposed the alternative approach that part of the nutrients must be dedicated to the basic functioning of the cells, implying that at low nutrient concentrations the cells do not grow, even if nutrients are still consumed [33]. Since, in our approach, low nutrient intakes result in very low growth rates, we consider the two approaches to be equivalent. In all the cases of this study, it was set to $v_{eff}=0.9.\;\mu_{i}(x,y,z,t)$ is the amount of nutrients consumed by bacterial cell i that depends on position (x,y,z) and time *t* of bacterium i. Nutrient uptake is calculated for each bacterium via Monod equation [34],


(2.2) $$\mu_{i}(x,y,z,t)=\frac{\mu_{max}\cdot C(x,y,z,t)}{K_{s}+C(x,y,z,t)}L_{i}^{*},$$

where $\mu_{max}$ denotes the maximum uptake rate per unit length, with a value in all cases of 1.1. $K_{s}$ is the nutrient concentration at which $\mu_{i}$ becomes $0.5\mu_{max}L_{i}^{*}$. $K_{s}$ provides a threshold below which the growth rate is conditioned by the concentration of nutrients. Nutrient concentration C(x,y,z,t) depends at each instant on the position. Nutrient concentration decreases because uptake by bacterial cells is compensated by diffusion from areas with higher concentration. Accordingly, the evolution of nutrient concentration is described by the following diffusion equation:


(2.3) $$\frac{\partial C(x,y,z,t)}{\partial t}=D_{N}{▽}^{2}C(x,y,z,t)-\sum \mu_{i}(x,y,z,t),$$

$D_{N}$ being the nutrient diffusion coefficient. Diffusion inside biofilm microcolonies is limited by the presence of the extracellular polymeric matrix [35]. Accordingly, a larger diffusion coefficient is expected in the bulk medium than within the colonies. However, when using a single $D_{N}$ value in the simulation, we observed that nutrient concentration was maximal (i.e. $C=C_{0}$) for the complete bulk medium volume, including the outer boundary of the colonies, for the duration of the simulations (see below). In contrast, nutrients were progressively depleted inside the colonies, indicating that diffusion does not limit local nutrient concentration at any of the $D_{N}$ values chosen unless the nutrients are actively consumed by the cells. We appreciate that introducing a separate, greater $D_{N}$ value for the bulk medium would lead to equivalent results while unnecessarily complicating the calculations. Therefore, for the sake of simplicity, we chose not to establish separate $D_{N}$ values for the bulk medium and the biofilm colonies. This equation has been numerically solved at each instant. To this end, the simulation box has been discretized by dividing it into cubic compartments of dimensions 10.85σ×10.85σ×10.85σ. This approximation assumes that the nutrient concentration inside each compartment is uniform. The sum in the second term on the right-hand side of the equation is over all bacterial cells within the compartment centred at position (*x*, *y*, *z*, *t*). Accordingly, the combination of equations (2.1) and (2.2) results in identical growth rate for all cells in the same compartment. As an initial condition in the solution of equation (2.3), at t=0, the concentration of nutrients in the nutrient solution is homogeneous and equal to $C_{0}$. As the boundary condition, $C_{0}$ remains constant over the time far away from the biofilm, while at z=0 on the substrate, reflecting boundary condition has been implemented (dC(x,y,z,t)/dz=0atz=0∀x,y,t).

The movement of bacterial cells has been modelled by means of a Brownian dynamics algorithm. Here, we assume that each individual particle is displaced due to the interaction with other particles and the substrate, but also due to thermal agitation. Such movement is described according to the following set of equations:


(2.4) $$\begin{array}{r} r_{i}^{∥}(t+\Delta t)=r_{i}^{∥}(t)+\frac{D_{i}^{∥}}{k_{B}T}F_{i}^{∥}(t)\Delta t+\sqrt{2D_{i}^{∥}\Delta t}R^{∥}{\hat{e}}_{i}(t) \end{array},$$

(2.5) $$r_{i}^{⊥}(t+\Delta t)=r_{i}^{⊥}(t)+\frac{D_{i}^{⊥}}{k_{B}T}F_{i}^{⊥}(t)\Delta t+\sqrt{2D_{i}^{⊥}\Delta t}\left[R_{1}^{⊥}{\hat{v}}_{i,1}(t)+R_{2}^{⊥}{\hat{v}}_{i,2}(t)\right],$$

(2.6) $${\hat{e}}_{i}(t+\Delta t)={\hat{e}}_{i}(t)+\frac{D_{i}^{\vartheta }}{k_{B}T}T_{i}(t)\times {\hat{e}}_{i}(t)\Delta t+\sqrt{2D_{i}^{\vartheta }\Delta t}\left[R_{1}^{\vartheta }{\hat{v}}_{i,1}(t)+R_{2}^{\vartheta }{\hat{v}}_{i,2}(t)\right],$$

where ${\text{r}}_{i}$ represents the trajectory of each particle i and ${\hat{e}}_{i}$ the orientation of its longitudinal axis. The terms ${\text{r}}_{i}^{∥}$ and ${\text{r}}_{i}^{⊥}$ denote the parallel and perpendicular projections of such trajectory, whereas ${\hat{v}}_{i,1}$ and ${\hat{v}}_{i,2}$ are two instantaneous vectors perpendicular to ${\hat{e}}_{i}$. $R^{∥}$, $R_{1}^{⊥}$, $R_{2}^{⊥}$, $R_{1}^{\vartheta }$ and $R_{2}^{\vartheta }$ are independent Gaussian random numbers of variance 1 and 0 mean. $D_{i}^{∥}$, $D_{i}^{⊥}$ and $D_{i}^{\vartheta }$ are the instantaneous values of the parallel, perpendicular and orientational short times diffusion coefficients of particle i. They depend on the dimensions of the particle, being calculated with the expressions


(2.7) $$\begin{array}{l} D_{∥}/D_{0}=-0.0198\cdot ln(L^{*})+0.0777+\frac{0.0437}{L^{*}}-\frac{0.0158}{L^{*2}}\\ D_{⊥}/D_{0}=-0.0119\cdot ln(L^{*})+0.0452+\frac{0.0796022}{L^{*}}-\frac{0.0190}{L^{*2}}\\ D_{i\vartheta }\sigma^{2}/D_{0}=-0.0002\cdot ln(L^{*})+0.0012-\frac{0.0243}{L^{*}}+\frac{0.3232}{L^{*2}}+\frac{0.2597}{L^{*3}}-\frac{0.0483}{L^{*4}}-\frac{0.1931}{L^{*5}} \end{array}.$$

These diffusion coefficients were calculated by a similar method to that proposed by Bonet Avalos *et al.* [36]. Here, $D_{0}=D_{0}^{*}\cdot \sigma^{2}/\tau$, where τ is the time unit and $D_{0}^{*}$ has been set to 0.1, as in previous studies [17,19,30].

${\text{F}}_{i}^{∥}(t)$, ${\text{F}}_{i}^{⊥}(t)$ and ${\text{T}}_{i}(t)$ are the parallel and perpendicular components of the forces and the torque of the forces over particle i, respectively. The origin of these forces and torques is the interaction of particle i with the rest of the particles, and the interaction between particles and substrate. The interaction with other particles is modelled with the truncated and shifted Kihara potential [37,38],


(2.8) $$U_{ij}\;=\left\{ \begin{array}{cc} 4\epsilon_{BB}\left[{\left(\frac{\sigma }{d_{m}}\right)}^{12}-{\left(\frac{\sigma }{d_{m}}\right)}^{6}+0.0154\right] & \;\;d_{m}\leq 2\sigma \\ 0 & \;\;d_{m}>2\sigma , \end{array}\right.$$

where i and j are two generic particles, and $d_{m}$ denotes the minimum distance between them. Details on the computation of the minimum distance between two spherocylinders can be found in [39]. $U_{ij}$ mimics the steric repulsion between particles at short distance, while it introduces an attraction for non-overlapping short distances. As explained in [40], forces and torques are easily obtained from this potential. The intensity of the attraction has been fixed as $\epsilon_{BB}=\epsilon$, with ϵ as the energy unit.

For interaction between particles and substrate, we have followed a more elaborate strategy. For each particle, we have estimated the shortest distance to the substrate surface, $d_{i,w}$. This is calculated as the distance from the central segment of the cylindrical part of the particle to the flat substrate. If this distance is shorter than $d_{rep}=(2/5{)}^{1/6}\sigma_{w}$, the particle i is subjected to a repulsive force ${\text{F}}_{i,rep}$ due to steric interactions, calculated with the following expression:


(2.9) $$F_{i,rep}=\epsilon_{rep}\cdot d_{i,w}\left[\frac{6}{5}{\left(\frac{\sigma_{w}}{d_{i,w}}\right)}^{10}-3{\left(\frac{\sigma_{w}}{d_{i,w}}\right)}^{4}\right]{\hat{e}}_{k}$$

$\epsilon_{rep}$ and $\sigma_{w}$ being two parameters that control the strength and range of this repulsive interaction. ${\hat{e}}_{k}$ is an unitary vector normal to the substrate (see figure 1*b*). This force, which is applied to each particle at the position closest to the substrate, derives from a repulsive interaction potential that has been used in the past by other authors to model the repulsive interaction of a substrate with molecular liquids [41,42].

Additionally, if the shortest distance to the substrate surface $d_{i,w}$ is less than $d_{c}>\sigma_{w}$, we have assumed an attractive interaction between particle and substrate. This interaction models the effect of the interaction of adhesion factors (surface proteins and polysaccharides) exposed on the cell surface with the substrate. We have assumed that each cell has a given number of such factors uniformly exposed on the cell surface. Adhesion factors are assumed to attach to the substrate, acting as a uniformly distributed set of springs. Adding up such contribution, we obtain the following expression for the net attractive force over a generic particle i:


(2.10) $${\text{F}}_{i,att}=-\epsilon_{att}L_{i}^{*}r_{z}(i){\hat{e}}_{k}$$

applied at the point ${\text{r}}_{p}=L_{i}^{*2}/(12r_{z}(i))\left({\hat{e}}_{i}\cdot {\hat{e}}_{k}\right)({\hat{e}}_{i})$. Here, $r_{z}(i)$ is the distance from the centre of the particle to the substrate. $\epsilon_{att}$ is a parameter that controls the strength of the attractive interaction. With this, the total force and torque over each particle due to the interaction with the substrate is the sum of the described repulsive and attractive contributions. The parameters of the interaction between particles and substrate have been fixed after several trials, looking for a similarity in the aspect of the biofilms obtained by applying the simulation algorithm with experimental colonies, as $\epsilon_{rep}=100\epsilon$, $\sigma_{w}=(2/5{)}^{1/6}\sigma$, $d_{c}=\sigma_{w}+0.5\sigma$ and $\epsilon_{att}=20\epsilon$. A representation of the distances involved in the calculation of the described interactions is shown in figure 1*b*.

With the algorithm described above, we have simulated the evolution of biofilms under different conditions starting from a single particle initially placed on the substrate. An example of a sequence of snapshots along the biofilm growth is shown in figure 1. In order to characterize the evolution of the shape, geometrical properties and internal structure of the biofilm colony, some observables have been calculated from simulations carried out with the method described in this section. Typically, the values obtained have been calculated by averaging over 30 independent simulation runs for each case. For instance, to characterize the size of the colony, we have estimated the amount of biomass in a given instant, m(t), as


(2.11) $$m(t)=\sum_{i=1}^{N(t)}L_{i}^{*}(t),$$

N(t) being the number of cells in the colony and $L_{i}^{*}(t)$ the aspect ratio of cell i at instant t.

To determine the geometry of the colony, we determined the ellipsoid that best fits the particle distribution. In this calculation, the colony was assumed to be half of an ellipsoid symmetric with respect to the z=0 plane. Thus, we have defined a number of points ($20L_{i}^{*}(t)$ per cell) randomly situated along the longitudinal axis of each bacterium. The components of inertia tensor have been calculated as $I_{\alpha ,\beta }=1/N_{p}\sum_{i=1}^{N_{p}}\left(\delta_{\alpha ,\beta }(\sum_{k=\alpha ,\beta }(r_{i}^{k}{)}^{2})-r_{i}^{\alpha }r_{i}^{\beta }\right)$. Here, the sum is over all the $N_{p}$ points generated at each instant. α and β indicate the coordinates x, y and z, $\delta_{\alpha ,\beta }$ is the Kronecker delta, and $r_{i}^{x}$ and $r_{i}^{y}$ are the corresponding x or y coordinates of the vector from the centre of mass of the colony to the position of the generated point i. $r_{i}^{z}$ is the *z* coordinate of the vector position of the point i. To take into account the symmetry of the problem, we have supposed that $I_{x,z}=I_{y,z}=0$. Diagonalizing this tensor it is possible to calculate the length and direction of three semi-axes, a>b in the plane xy, and c in the direction z, of the ellipsoid that best fits the distribution of cells in the biofilm colony [43]. From these three semi-axes, we have estimated the volume ($V_{e}=2/3\pi abc$) and the external surface ($A_{e}$) of the colony as half of those of the ellipsoid. For $A_{e}$, we have used the expression proposed by Minchon [44]. We have also calculated the area of the maximum ellipse, where the ellipsoid resulting from the described fit intersects with the z=0 plane, $A_{z}=\pi /4ab$. This last surface corresponds to the area where the colony rests on the substrate.

From the geometrical parameters of the resulting ellipsoid, it is also possible to obtain other values to characterize the properties of the biofilm colonies. For example, as a measure of the compactness of the colony, we have calculated the instantaneous value of the density, defined as


(2.12) $$\rho (t)=\frac{m(t)}{V_{e}}.$$

To analyse the shape of the colonies, we have defined a triaxial parameter $T_{p}$ as


(2.13) $$T_{p}=\frac{a^{2}-b^{2}}{a^{2}-c^{2}}.$$

This parameter gives information about the triaxial character of the fitted ellipsoid, and therefore of the colonies. If b=c (then $T_{p}=1$), a=c ($T_{p}=\infty$) or a=b ($T_{p}=0$), the ellipsoid is biaxial. In other cases, with the exception of the trivial case a=b=c ($T_{p}=1$), the ellipsoid is triaxial with three different semi-axes. To obtain additional information about the shape of the colonies, we have also defined a biaxial parameter $B_{p}$, that indicates how similar the elliptical base of a colony is to a circumference. It is defined as


(2.14) $$B_{p}=\frac{a^{2}-b^{2}}{a^{2}+b^{2}}.$$

We also have explored the orientational correlations inside the biofilm colonies. Thus, we have calculated the nematic order parameter $S_{2}(t)$. $S_{2}(t)$ is calculated with the standard procedure of diagonalizing a symmetric tensor traceless build with the orientation vectors of all the particles [45]. $S_{2}(t)$ provides information about the global orientational correlation inside the biofilm colonies. To complete this information, we have also calculated the local orientational order. To this end, at a given instant, we obtained the orientational distribution function between two particles. This function is defined as the average for each intermolecular distance of the second Legendre polynomial of the dot product between the orientation vector of cells i and j at instant t, $g_{2}(r)=\left\langle P_{2}({\hat{e}}_{i}(t)\cdot {\hat{e}}_{j}(t))\right\rangle .$ This function provides information on the distance dependence of the averaged relative orientation between the particles, being useful for evaluating the existence and the size of possible nematic domains.

## 3. Results

We have used the algorithm described above to explore the impact of nutrient distribution and the sensitivity of bacteria to the concentration of nutrients in the environment on the characteristics of the biofilm throughout its growth. Taking into account that $K_{s}$ represents the sensitivity of bacteria to nutrient limitation and $C_{0}$ is the maximum nutrient concentration, we have defined $K_{s}^{*}=K_{s}/C_{0}$. We have set this parameter to three different values, one ($K_{s}^{*}=10$) representing severe nutrient limitation, one representing mild nutrient excess ($K_{s}^{*}=0.7$) and one representing large nutrient excess ($K_{s}^{*}=0.01$). In addition, as the actual nutrient concentration at each point is the balance between nutrient uptake and diffusion, we have set two conditions representing low ($D_{N}^{*}=D_{N}/D_{0}=100$) and high values ($D_{N}^{*}=1000$) of the nutrient diffusion coefficient.

**Figure 2.**
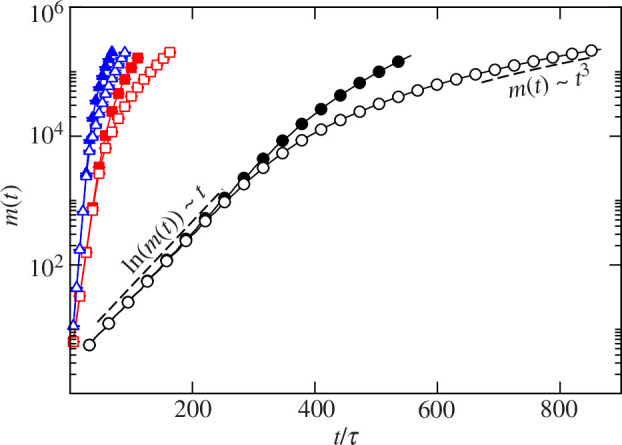
Biomass of the biofilm colony m(t) as a function of time t/τ. Black curves and circles $K_{s}^{*}=10$, red curves and squares $K_{s}^{*}=0.7$, blue curves and triangles $K_{s}^{*}=0.01$. Solid and open symbols $D_{N}^{*}=1000$ and 100, respectively.

As a first approach, we calculated the evolution of the total biomass of the biofilm (m(t)) under all combinations of the $K_{s}^{*}$ and $D_{N}$ parameters (figure 2). The change in biomass over time proved to be very sensitive to both parameters. Accordingly, the increase in biomass was inversely correlated with $K_{s}^{*}$. For all values of $K_{s}$, at short times, biomass increased exponentially over time, regardless of the value of the nutrient diffusion coefficient. In this regime and for all cases, the population growth rate takes the maximum value of the growth rate obtained from equations (2.1) and (2.2). Since this maximum rate is achieved when $C(x,y,z,t)=C_{0}$, such exponential growth suggests that all cells in the biofilm have full access to nutrients at this stage.

At intermediate times, biomass growth slows down. At this stage, there is a direct correlation between the increase in m(t) and the value of $D_{N}$. Interestingly, at the condition representing severe nutrient limitation ($K_{s}^{*}=10$) and low nutrient diffusion ($D_{N}/D_{0}=100$), m(t) grows as $t^{3}$ at the end of the simulation period. This behaviour is expected if biomass growth were proportional to the colony surface area,. Thus, we hypothesize that in the later stages of biofilm evolution, the non-uniform nutrient distribution may restrict bacterial growth to the outer surface of the colony. While our simulation set-up only detects this behaviour at low nutrient concentration and diffusion (i.e. most severe nutrient limitation conditions), we presume that it may be observed in other conditions with longer simulation times.

**Figure 3.**
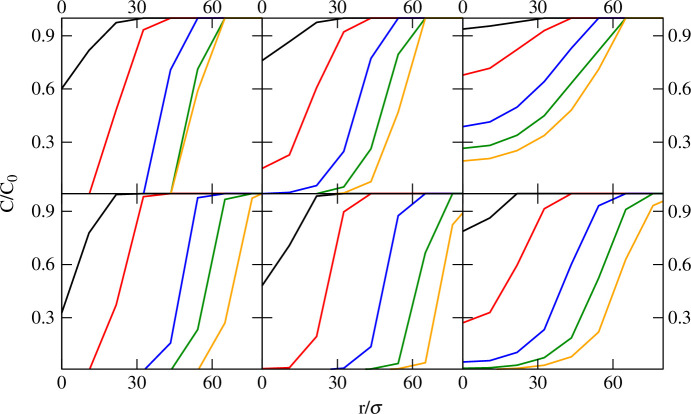
Nutrient concentration at a distance r/σ from the centre of mass of the biofilm colony in a layer adjacent to the substrate. Black, red, blue, green and orange curves correspond to biomass $m(t)=10^{3},10^{4},5\times 10^{4},10^{5}$ and $1.5\times 10^{5}$, respectively. The top row shows simulations with $D_{N}^{*}=1000$, bottom row shows simulations with $D_{N}^{*}=100$. Left, centre and right columns correspond to $K_{s}^{*}=0.01,0.7$ and 10, respectively. The relative error is in all cases is below 5%.

Next, the spatial distribution of nutrients was evaluated for various values of $K_{s}^{*}$ and $D_{N}$. Figure 3 represents the distribution of nutrient concentration in the layer adjacent to the substrate as a function of the distance r from the colony centre of mass. Our results show that, while $C=C_{0}$ at the outer boundary of the biofilm in all cases and at all times, biofilm growth leads to progressive nutrient depletion in the central core of the colony. This effect is least evident at $K_{s}^{*}=10$ and $D_{N}^{*}=1000$, i.e. high nutrient diffusion and poor nutrient uptake (figure 3, top right). At decreasing $D_{N}^{*}$ and $K_{s}^{*}$ values, depletion becomes more apparent to the point that a sharp decline in concentration at the outer colony boundary leads to undetectable nutrient concentration at the colony central core even at relatively low biomass values, especially under a high nutrient uptake regime ($K_{s}^{*}=0.01$) (figure 3, top and bottom left). The decrease in nutrient concentration observed here supports the notion expressed above that, as nutrients become depleted, cell growth and proliferation is arrested at the inner core and restricted to cells at the outermost layers of the colony.

**Figure 4.**
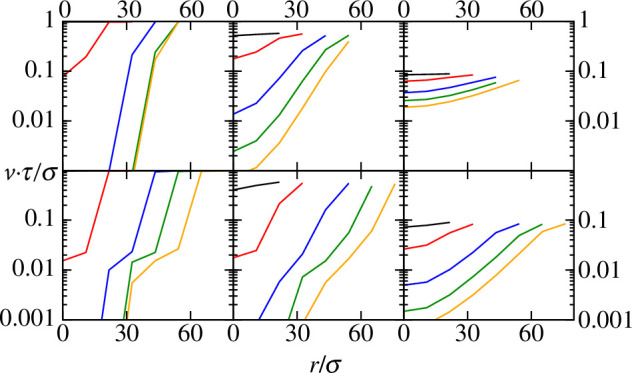
Averaged bacterial growth rate at a distance r/σ from the centre of mass of the biofilm in a layer adjacent to the substrate. Black, red, blue, green and orange curves correspond to biomass $m(t)=10^{3},10^{4},5\times 10^{4},10^{5}$ and $1.5\times 10^{5}$, respectively. The top row shows simulations with $D_{N}^{*}=1000$, bottom row shows simulations with $D_{N}^{*}=100$. Left, centre and right columns correspond to $K_{s}^{*}=0.01,0.7$ and 10, respectively. The relative error is in all cases is below 5%.

Direct analysis of position-dependent growth rates reveals that the non-homogeneous concentration of nutrients indeed has direct consequences on bacterial growth within the biofilm colony, as shown in figure 4. Accordingly, low values of $K_{s}^{*}$ result in limited or absence of bacterial growth at the inner core of the colony. Although low values of $K_{s}^{*}$ correlate with fast nutrient uptake and potentially faster growth even at low nutrient concentrations, nutrient depletion due to fast uptake at the colony inner core not balanced by diffusion leads to a decline in growth rate that drops to 0 at large biofilm biomass. In contrast, in the outermost layers of the colony (large r/σ values), which are in direct contact with the bulk medium, nutrient uptake is balanced by diffusion and the growth rate remains effectively high. In contrast, under poor uptake regimes ($K_{s}^{*}=10$), nutrients are not depleted (or depletion is much slower) and growth is not arrested in the colony core region. This behaviour is observed both for low and high $D_{N}^{*}$ conditions (figure 4, left column).

**Figure 5.**
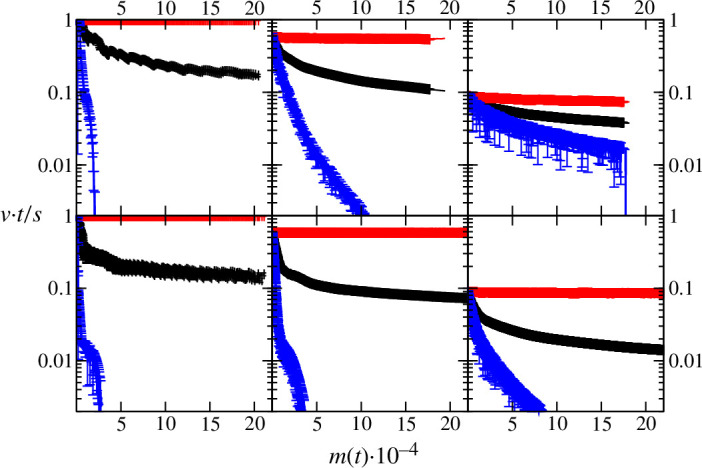
Maximum (red), averaged (black) and minimum (blue) growth rates in the biofilm as a function of the amount of biomass m(t). The top row shows simulations with $D_{N}^{*}=1000$, bottom row shows simulations with $D_{N}^{*}=100$. Left, centre and right columns correspond to $K_{s}^{*}=0.01,0.7$ and 10, respectively.

Similar conclusions can be drawn from the analysis of maximum, average and minimum growth rates as a function of biofilm biomass (figure 5). In each of the conditions explored, the maximum growth rate is essentially constant, reflecting the growth of bacteria at the outer layers of the biofilm colonies, i.e. with full access to nutrients in the bulk medium. The maximum growth rate increases with decreasing values of $K_{s}^{*}$, as can be calculated from the equations (2.1) and (2.2). In contrast, the minimum growth velocity evolves with increasing biomass showing different patterns depending on the $K_{s}^{*}$ values. Specifically, at $K_{s}^{*}=0.01$ the minimum growth velocity drops to 0 even at low biomass values. As explained above, this is probably because of the local growth arrest due to fast nutrient depletion at the inner core of the colony. In contrast, at $K_{s}^{*}=10$ the minimum growth velocity does not drop to 0 ($D_{N}^{*}=1000$), i.e. growth is not arrested in any region of the biofilm or it only drops to 0 at greater biomass values ($D_{N}^{*}=100$). For $K_{s}^{*}=0.7$, an intermediate outcome between the $K_{s}^{*}=0.01$ and 10 conditions is achieved. The dependence of growth rates on the combination of $D_{N}^{*}$ and $K_{s}^{*}$ values highlights the notion that local nutrient concentration, and eventually local bacterial growth, are driven by the local balance of nutrient uptake and diffusion.

**Figure 6.**
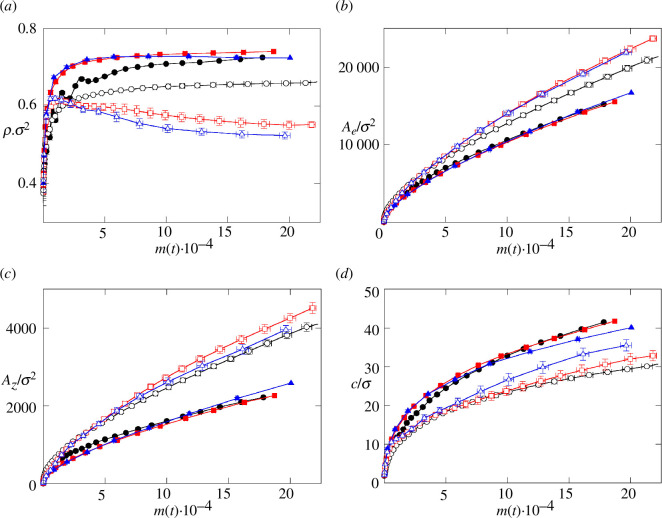
Geometrical properties of the colony. (*a*) Evolution of the density in reduced units $\rho \sigma^{2}$, (*b*) external surface of the biofilm in reduced units $A_{e}/\sigma^{2}$, (*c*) biofilm base area in reduced units $A_{z}/\sigma^{2}$ and (*d*) biofilm height in reduced units c/σ, as a function of the biomass m(t). Symbols and colours represent the same cases as in figure 2.

We have also explored the impact of the non-homogeneous distribution of nutrients and cell growth described above on the properties and structure of the growing biofilm. Figure 6*a* shows the evolution of density ρ(t) with biomass for the different conditions explored in this work. Biomass density, which is inversely proportional to colony volume is indicated in equation (2.12), increases very fast at the early growth stages, when m(t) values are very low. However, in later stages, the increase in density slows down, and shows somewhat different behaviours as a function of $K_{s}^{*}$ and $D_{N}^{*}$. Hence, at $D_{N}^{*}=1000$ (solid symbols in figure 6), ρ increases more slowly than at $K_{s}^{*}=10$ than at $K_{s}^{*}=0.7$ and 0.01. At the final steps of the simulation, density still increases with biomass at $K_{s}^{*}=10$, while it reaches a plateau at $K_{s}^{*}=0.7$ and declines at $K_{s}^{*}=0.01\rho$. At $D_{N}^{*}=100$ (open symbols) ρ does not cease to increase with increasing m(t) at $K_{s}^{*}=10$, while at $K_{s}^{*}=0.7$ and 0.01 and $m(t)>3\times 10^{3}$, ρ decreases with m(t). In this range, for any given value of m(t)ρ, *ρ* increases with $K_{s}^{*}$ values.

Density, which is a proxy of colony compactness, is closely related to the distribution of nutrients within the biofilm and established growth regimes. Thus, as reflected in figures 3–5 and discussed above, for $K_{s}^{*}=10$ nutrients are not completely depleted at the colony core, and therefore growth is not arrested. Such uninterrupted cell growth, even at a low rate, leads to a sustained increase in pressure at the inner core of the colony [18], which favours an increase in density. Conversely, as at $K_{s}^{*}=0.7$ and 0.01 nutrient depletion and cell growth arrest prevents pressure build-up within the colony. Hence, the dependence of ρ on nutrient diffusion is a direct consequence of the features observed and discussed above (figures 3–5).

We have also explored geometrical aspects of the biofilm colonies as they relate to the spatial distribution of nutrients in the conditions described above. Figure 6*b* depicts the evolution of the external surface area of the biofilm colony ($A_{e}$) with biomass for different values of $D_{N}^{*}$ and $K_{s}^{*}$. Similarly, figure 6*c* shows the evolution of the biofilm base area ($A_{z}$), while figure 6*d* presents the biofilm colony height (c). These three magnitudes are extracted from the ellipsoid that best fits the cell distribution, as described in the Methods section. In all cases, the three magnitudes increase with biomass, reflecting colony expansion with cell growth. This increase is highly modulated by the value of $D_{N}^{*}$ and, consequently, by the distribution of nutrients within the colony. Accordingly, figure 6*b* shows how lower nutrient diffusion is associated with larger external surface of the colony, $A_{e}$. Similarly, figure 6*c* indicates that $A_{z}$ increases with decreasing $D_{N}^{*}$ values for a given value of biomass. Conversely, from figure 6*d*, colony height shows the opposite behaviour, as c is increased at a higher diffusion coefficient.

The behaviours described for $A_{e}$, $A_{z}$ and c suggest that low nutrient diffusion favours flattened colonies displaying a larger contact surface with the substrate, as well as a larger external surface. A larger outer surface of the biofilm favours the access of more cells to the nutrient pool. Since low diffusion favours nutrient depletion inside the colony, restricting cell growth to the outer surface, a flat colony architecture is expected to favour colony growth under such conditions. Because cell regulation mechanisms are not taken into account in this study, this adaptation of biofilm morphology to nutrient diffusion is a direct result of the physical interaction of the components in the system.

**Figure 7.**
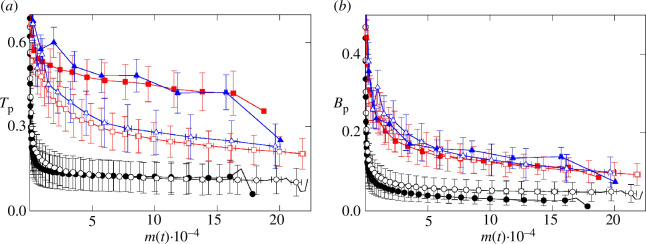
Analysis of colony shape. (*a*) Evolution of triaxial parameter $T_{p}$ and (*b*) biaxial parameter $B_{p}$ as a function of the biomass m(t). Symbols and curves are for the same cases as in figure 2.

Additional information about the evolution of the geometrical features of the biofilm colonies can be drawn from the triaxial ($T_{p}$) and biaxial ($B_{p}$) parameters (figure 7). In all the simulation conditions, the triaxial parameter $T_{p}$ (figure 7*a*) ranges between 0 and 1, indicating that colony height is between the size of the other semi-axes, this is a>c>b. In addition, $T_{p}$ decreases with biofilm growth, especially at the earlier stages. A similar behaviour is observed for the biaxial parameter (figure 7*b*). These observations strongly suggest that biofilm colonies evolve from an ellipsoidal shape towards a spherical shape. The decrease in $T_{p}$ and $B_{p}$ is more pronounced for $K_{s}^{*}=10$ than for $K_{s}^{*}=0.7$ and 0.01. This differential behaviour may be due to the slower biomass growth at low $K_{s}$ (see figure 2), suggesting that prevalence of cell diffusion over cell growth may lead to the formation of more homogeneous and circular structures, as previously shown for two-dimensional biofilm colonies [17,19].

**Figure 8.**
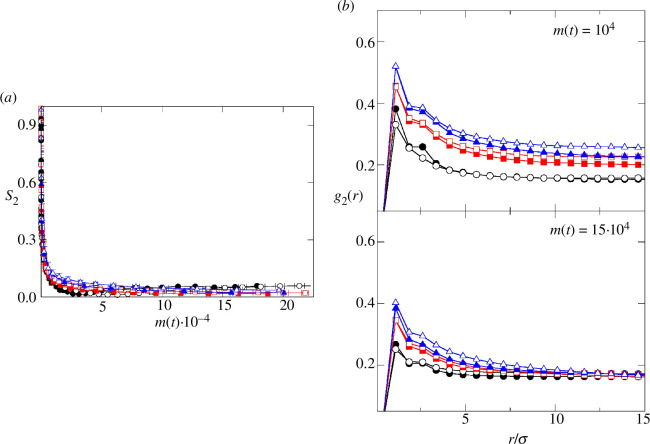
Orientational correlations within the colony. (*a*) Evolution of the nematic order parameter $S_{2}$ as a function of the biomass m(t). (*b*) Orientational correlation function $g_{2}(r)$. Top panel corresponds to $m(t)∼10^{4}$ and the bottom panel corresponds to $m(t)∼15\times 10^{4}$.Symbols and curves are for the same cases as in figure 2.

It is interesting to explore the global orientational correlation between the cells. Thus, as figure 8*a* shows how, for all cases, no global nematic order is observed in the colony from early developmental stages. Accordingly, at $m(t)>5\times 10^{4}$ the nematic order parameter drops to values very close to 0. In contrast, we have detected orientational correlation between particles as a function of the distance between them. Thus, for low values of biomass ($m(t)=10^{4}$, top panel of figure 8*b*) and low $K_{s}^{*}$, the orientational distribution function $g_{2}(r)$ has values greater than 0.3 up to significant distances. This is consistent with the occurrence of nematics domains with size 10σ. For $K_{s}^{*}=10$, the orientational distribution function drops faster, indicating the existence of smaller nematic aggregates. These findings are consistent with our previous results [17,19], where the parameter Γ (which measures the ratio between bacterial cell growth and diffusion) was shown to control local orientational order in two-dimensional biofilm colonies. In that study, high Γ values; i.e. cases where growth predominates over diffusion, were associated with the appearance of local domains. Conversely, low Γ was related to disordered colonies. Here, low $K_{s}^{*}$ conditions at this stage are related to faster growth and hence can be extrapolated to high Γ, leading to the formation of well-defined domains. On the other hand, $K_{s}^{*}=10$ implies slower average bacterial growth (which can be assimilated to lower Γ), consistently resulting in more disordered colonies. Simultaneously, all studied cases measured at higher biomass levels ($m(t)=15\times 10^{4}$, bottom panel of figure 8*b*) show lower values of $g_{2}(r)$ at all distances, indicating a lower level of orientational correlation. This can be rationalized as a consequence of the lower concentration of nutrients within the biofilm at later stages of development. In this situation, slow cell growth leads to the predominance of cell diffusion, disfavouring the correlational orientation of the biofilm cells.

## 4. Conclusions and final remarks

In this study, we present a computer simulation methodology to study the evolution and characteristics of three-dimensional biofilms. The method used is an extension of the two-dimensional methodology developed and used in previous works [17,19,30]. In the version used here, in addition to considering the transition from the initial two-dimensional colony to a three-dimensional biofilm, we have introduced the coupling of the bacterial growth rate, and consequently biofilm growth, with local nutrient concentration resulting from the balance between diffusion and uptake by the growing cells.

The results obtained with our simulation algorithm confirm that the coupling of bacterial growth with nutrient uptake leads to a stratification of biofilm colonies, whereby nutrients are depleted at the inner core of the colony, resulting in a local arrest of growth and proliferation. Our model predicts that the increase in biomass at this point is owing to cell growth at the outermost layers of the biofilm. Similarly to tumour growth, biofilm colony stratification due to nutrient uptake and diffusion limitation has been reported previously by other studies [35,46,47]. Our work joins this previous evidence with the conclusion that this stratification is more evident the more efficient the bacteria are at nutrient uptake, as reflected by a smaller value of the $K_{s}$ parameter of the Monod equation (equation 2.2), which implies high uptake rates even at low nutrient concentrations, leading to increased growth rates. In these conditions, we observe that high nutrient uptake leads to fast nutrient depletion at the inner core of the biofilm colony. Accordingly, starvation leads to an arrest of cell growth in this region. Conversely, bacteria that are less efficient at consuming nutrients do not fully deplete them (or take longer to do so), and therefore do not completely stop their growth at the core regions (or only stop growth at later stages). Our results also suggest that pressure build-up due to fast cell growth inside the colony at lower $K_{s}$, results in more compact colonies. This is an aspect on which we are developing further studies.

Another interesting result is that biofilms in which bacteria exhaust nutrients faster tend to form colonies with larger external surfaces. This favours an increase in the fraction of cells close to the surface. Access of these cells to nutrients in the bulk medium allows them to support the growth of the colony. This response of the cell colony morphology to nutrient depletion at the inner core is solely a consequence of physical interactions between the components of the system in the absence of any genetic or physiological control of the cell behaviour.

Our model assumes that nutrient diffusion in the bulk medium is equivalent to that occurring inside the colony structure. While this assumption is unrealistic because it does not reflect the known limitations to diffusion within the biofilm polymeric matrix [35], we found that nutrient concentration at the outer boundary of the biofilm was homogeneous with that in the bulk medium volume in all our simulations. Therefore, it is our understanding that this simpler approach does not introduce any artefacts that compromise its validity for the study of the establishment of nutrient gradients inside the biofilm.

Our model also predicts that other geometric and structural properties of the biofilm are also modulated by the balance between nutrient uptake and diffusion, including biofilm colony shape and orientational correlation between cells. Our observations confirm and extend the model previously described for two-dimensional biofilm growth [17], in which the balance between cell growth and diffusion decisively determines multiple geometrical parameters of biofilm colony growth. In conclusion, the model presented here supports a number of interesting predictions regarding growth, nutrient distribution and internal organization of growing bacterial biofilms. We believe that the algorithm we present, and cell simulation in general, can be a powerful tool for the study of numerous aspects of the growth and development of bacterial biofilms.

## Data Availability

Supplementary material available online at [48].
